# Silica-Containing Biomimetic Composites Based on Sea Urchin Skeleton and Polycalcium Organyl Silsesquioxane

**DOI:** 10.3390/biomimetics8030300

**Published:** 2023-07-09

**Authors:** Nikolay P. Shapkin, Irina G. Khalchenko, Anatoliy L. Drozdov, Aleksander N. Fedorets, Igor Yu Buravlev, Anna A. Andrasyuk, Natalya V. Maslova, Kirill A. Pervakov, Evgeniy K. Papynov

**Affiliations:** 1 Far Eastern Federal University, 10 Ajax Bay, Russky Island, 690922 Vladivostok, Russia; shapkin.np@dvfu.ru (N.P.S.); khalchenko.ig@dvfu.ru (I.G.K.); fedorec.an@dvfu.ru (A.N.F.); andrasyuk.aa@students.dvfu.ru (A.A.A.); maslova.nv@dvfu.ru (N.V.M.); pervakov.ka@students.dvfu.ru (K.A.P.); papynov@mail.ru (E.K.P.); 2 A.V. Zhirmunsky National Scientific Center of Marine Biology, Far Eastern Branch of the Russian Academy of Sciences, St. 17 Palchevsky, 690041 Vladivostok, Russia; anatoliyld@mail.ru

**Keywords:** biomimetic composite, polycalciumethylsilsesquioxane, polycalciumphenylsilsesquioxane, sea urchin skeleton, coherent scattering region

## Abstract

The paper presents an original approach to the synthesis of polycalciumorganyl silsesquioxanes through the reaction of polyorganyl silsesquioxanes [*R*SiO_1.5_]_n_ (where *R* is an ethyl and phenyl radical) with sea urchin skeleton under the conditions of mechanochemical activation. The novelty and practical significance of the present study lies in the use of an available natural raw source as a source of calcium ions to initiate the reaction of calcium silicate formation and create a matrix for the formation of a porous inorganic composite framework. The thermal stability of the introduced silicates, i.e., the ability to maintain a porous structure at high temperatures, is key to the production of an ordered porous material. The reaction scheme was proposed to be based on the interaction of calcium carbonate with the siloxane bond. FTIR, XRD, GPC, and TGA were used to study the composition and structure of the obtained materials. The cross-sectional area of the polymer chain and the volumes of the coherent scattering regions of the polymers obtained were calculated from the XRD data. To prepare the composites, the sea urchin skeleton was further modified with polycalciumorganyl silsesquioxanes in a toluene solution. To remove the sea urchin skeleton, the obtained biomimetic composites were treated with hydrochloric acid. The results of the morphological and surface composition studies are reported. The method proposed in the paper could be of fundamental importance for the possibility of obtaining structured porous composite materials for a wide range of practical applications, including for the purpose of creating a composite that may be a promising carrier for targeted delivery of chemotherapy agents.

## 1. Introduction

Composites based on biological materials (and their wastes) are capable of exhibiting multi-scale structural properties that are unparalleled in man-made materials, as demonstrated by advances in a wide range of scientific research over the last few decades [1]. The unique properties of natural materials are the result of evolutionary selection by living organisms over millions of years. They have been shaped by nature’s use of a rather limited chemical set. In this context, it is possible to develop high-performance materials with a range of unique functional properties by combining the evolutionary structural features of such natural materials with modern advances in the chemistry of synthetic systems. Optimal structural properties may vary depending on the internal chemistry and properties of the material’s basic constituents, as well as various requirements and constraints in natural environments and engineering applications. It is also expected that fundamentally new materials with low production costs will emerge from the synergistic effect of the cumulative integration of advances in each research area. At the same time, it is clear that characterization studies for the analysis of biological materials will continue to be based on experimental methods currently in use in other fields. Using methods capable of preserving and/or reproducing the thin, heterogeneous structures found in biological composites is certainly a challenge that needs to be addressed.

The class of biomimetic materials, which is considered a distinct and advanced field of materials science, consists of synthetic materials that can mimic the structural and functional properties of biological systems. As biomaterials for bone regeneration [2] and skin tissue [3], drug delivery agents [4], antibacterial systems [5], antioxidants [6], etc., biomimetic materials have demonstrated their excellent functional properties for addressing medical tasks. The demand for biomimetic materials to solve problems in the synthesis and fabrication of optical [7] and electrochemical [8] sensors, materials for energy sources (anode materials for lithium–ion batteries [9]), materials and products with increased corrosion resistance [10], triboelectric nanogenerators [11], and other devices from a wide range of technical fields [12], including electrochemical applications [13,14,15], is also evident from an analytical review of scientific and technological advances.

Sea urchins are considered a delicacy in many parts of the world and are commercially farmed in specialised facilities [16]. Depending on the species, the edible parts of sea urchins make up about 13–20% of their total weight, and the rest can be considered waste. After technologically simple processing, these wastes can be used as fillers or as animal feed and soil additives. However, nowadays, more efficient methods of waste processing are being studied due to their biological natural origin [17]. The chemical composition, biological origin, low cost, and wide availability around the world nominate sea urchins as a very promising source of biological material. 

Sea urchins should be mentioned as having rich potential for practical use in the obtention of biomimetic products, as they are sources of raw materials for organo–inorganic biocomposites [18,19]. Recently, sea urchins have been used to produce bioceramics for biomedical applications [20,21], including materials with high antibacterial activity [22]. Sea urchins belong to the echinoderm class of marine invertebrates, many with morphological and biochemical features that have high biomimetic potential [23]. In this context, they have been successfully used to produce biomimetic materials [24,25,26,27,28], materials for bone regenerative medicine [29], and also functional materials such as biodegradable heat-resistant films for food packaging uses [30,31]. Maddocks and Harris attempted to produce porous silicon carbide (SiC) by infiltrating porous stereomes from sea urchin shells [32]. Indeed, organisms are capable of producing crystals with high precision in shape and morphology using macromolecules that act as modifiers. An alternative strategy might be to extract crystals from biomineralization products. One paper [33] deals with waste products from mussel shells. They consist mainly of monocrystals of calcite fibres and aragonite pellets that form the outer and inner layers, respectively. The characterization of these monocrystals showed special properties compared to natural or synthetic calcium carbonate. These biomaterials have been used in the field of dye water purification and have demonstrated a high anionic dye adsorption capacity.

It should also be emphasised that biomimetics includes not only materials obtained by processing raw materials of biological origin but also materials that borrow (repeat) the features of their structure from them. For example, in [34], catalysts based on mesoporous copper and manganese oxides were synthesised; morphological characterisation showed that the primary catalyst particles were nanorods that aggregated together to form structures similar to sea urchins (which was a factor in the increase in specific surface area). Recently published research has also revealed an original and efficient approach to the synthesis of a material for the production of an anti-cancer drug delivery agent [35]. The material was obtained by hydrothermal treatment of sea urchin skeletons in a sodium metaxylate (Na_2_SiO_3_·5H_2_O) solution with further modification of the resulting product using the 5-fluorouracil (C_4_H_3_FN_2_O_2_) sorption saturation method.

Sea urchin skeletons are characterized by a complex bicontinuous porous structure, also known as a mechanical tissue system (stereome). The stereome often exhibits controlled gradients of porosity and structural changes [19]. At the same time, individual stereome-based skeletons are calcite-based monocrystals containing magnesium. This indicates that the inorganic base of the sea urchin skeleton is chemically active when used as a raw material in chemical synthesis [19,36]. The strength and other mechanical properties of sea urchin shells and spicules vary with the chemical composition and structural organization of their components [31,37]. Sea urchin shells and spicules are composed of a calcium–organic composite material with inlays of other metals (Mg, Fe, Zn and Rb). A spongy stereome of crystalline magnesian calcite with the formula MgO_0.6_CaO_0.94_CO_3_ forms the basis of the mineral skeleton of sea urchins. Sea urchins combine the strength and volume of their skeletons with a minimum of material, using calcium carbonate with extraordinary efficiency. In the skeleton of sea urchin spicules, magnesium is not distributed homogeneously: the inner parts of the spicules contain more of it than the outer parts. These differences correlate with the stiffness and hardness of the spicules. Thus, it is clear that the mechanical heterogeneities of the solid microporous material comprising the echinoderm skeletal elements may have adaptive functional significance [38].

Polycalcium organylsesquioxanes are rather difficult to synthesize using crystalline CaCO_3_ or CaO. One paper [39] describes the synthesis and use of polycalciumphenylsesquioxane. Sea urchin shell is a potential source of calcium for the synthesis of calcium-containing biomaterials, as the shell is mainly composed of calcium carbonate. The development of a new synthesis method using marine invertebrate skeletons and siloxanes is a very important task. The thermal stability of the introduced silicates, i.e., the ability to maintain a porous structure at high temperatures, is the key to producing an ordered porous material. Previously, using poly ferro phenyl siloxane and the skeleton of the sea urchin *Strongylocentrotus intermedius* as a structuring template, a method was proposed for the template synthesis of biomimetic porous composites [40]. The application of a variety of synthetic methods, ranging from sol–gel chemistry to chemical precipitation, allowed the synthesis of amorphous and polycrystalline solids. Depending on the type and amount of sediment, the structure could be controlled up to a perfect copy of the sea urchin plate. Polyorganylsilsesquioxanes (POSS) are a well-studied class of organosilicon compounds since they possess valuable physical and technical properties [41]. The formation of ladder POSS represents a rare example of the transformation of cage-like polycyclic structures into a ladder macromolecular chain. Along with ladder polyphenylsilsesquioxane, a series of ladder polyalkyl(aryl)silsesquioxanes was synthesized. 

Considering the possibilities for chemical and biochemical applications of sea urchin skeletons to fabricate new functional materials, in this paper, polycalcium organyl silsesquioxanes were obtained by reacting polyorganyl silsesquioxanes [*R*SiO_1.5_]_n_ (where *R* is an ethyl and phenyl radical) with sea urchin skeleton under mechanochemical activation. In order to determine the possibility of obtaining structured composites for wide practical applications, the method proposed in this paper may be of fundamental importance. The authors believe that the method developed in this study for synthesizing new functional biomimetic composites based on ecologically pure natural resources may have wide practical application.

## 2. Materials and Methods

Three synthetic steps were proposed to obtain silicon-containing biomimetic composites: (1) Preparation of polycalcium organylsesquioxane by mechanochemical activation; (2) Modification of the sea urchin skeleton surface with the obtained polycalcium organylsesquioxane; (3) Extraction of the sea urchin skeleton with hydrochloric acid. After treatment of the composites with hydrochloric acid, calcium carbonate is removed, but the layered structure of the composite is preserved. The research strategy is to obtain and isolate the maximum amount of polycalcium organylsiloxanes using the method of mechanochemical activation. The conditions of mechanochemical activation for obtaining different polycalcium organylsiloxanes are optimized and elaborated [42,43,44,45,46]. Due to the friable, permeable structure of the sea urchin skeleton, synthesis is much faster and yields higher yields than with crystalline CaCO_3_ or CaO [39].

### 2.1. Materials

This study used the intermediate (grey) sea urchin *Strongylocentrotus intermedius* collected at the Vostok Marine Biological Station of the A.V. Zhirmunsky National Research Centre for Marine Biology (Far East Branch of the Russian Academy of Sciences) in the Vostok and Ussuri Bays of the Peter the Great Gulf, the largest gulf in the Sea of Japan. The species *Strongylocentrotus intermedius* is widely known from the scientific literature. It has been studied for several reasons. First, Strongylocentrotus intermedius has a high nutritional value. It is rich in proteins, amino acids, and long chain polyunsaturated fatty acids [47]. Second, it is used for the assessment of its sensitivity to several common toxicants, such as heavy metal ions, pesticides, detergents, and water-soluble hydrocarbons from diesel fuel [48]. Third, the marine environment is considered to be one of the most important sources of natural bioactive compounds with an extremely rich biodiversity. Sea urchins, in particular *Strongylocentrotus intermedius*, represent a source for the extraction of anti-cancer compounds [49].

### 2.2. Synthesis Methods

#### 2.2.1. Synthesis of Polyethyl Silsesquioxane (PES)

A mixture of 200 mL of water and 100 mL of butyl alcohol was placed in a conical flask with a magnetic stirrer. The flask was then placed in an ice bath. From a dropping funnel, 57 mL of ethyltrichlorosilane (0.4281 mol, ρ = 1.23 g/mL) was added dropwise for 40 min with uniform stirring. Stirring was continued for a further 20 min. The mixture was then transferred to a separating funnel and toluene was added to the mixture as an extragent. The resulting mixture was washed with distilled water until the pH ≈ 7. Anhydrous granulated calcium chloride was used to dry the organic layer for several hours. Then, the mixture was filtered, the residual solvents removed in a rotary evaporator, and the resulting polymer dried to constant weight in a vacuum oven at 80 °C.

#### 2.2.2. Synthesis of Polyphenyl Silsesquioxane (PPhS)

The synthesis of PPhS was carried out using the method described in [50]. The data of the elemental analysis are given in Table 1.

#### 2.2.3. Synthesis of Polycalcium Ethylsilsesquioxane (PCaES)

PCaES was obtained through the interaction of the sea urchin skeleton and PES under conditions of mechanochemical activation. A quantity of 10 g of sea urchin ground in a mortar and 8.1 g of PES were placed in a Pulverisette 6 planetary mill (“FRITSCH”, Idar-Oberstein, Germany) for 6 min at an operating frequency of 10 Hz (600 rpm^–1^) with 8 mm diameter steel balls. The mass of the substance obtained was 16.87 g (93.2%). The substance obtained was then heated at 250 °C for 30 min; the mass was 16.21 g (89.5%). Extraction of product 1 with toluene for one hour isolated PCaES with a yield of 26%. The elemental analysis data are given in Table 2.

#### 2.2.4. Synthesis of Polycalcium Phenylsilsesquioxane (PCaPhS)

Synthesis of PCaPhS was carried out similarly to the synthesis of PCaES. The data of the elemental analysis are given in Table 2. 

#### 2.2.5. Synthesis of Composite 1

For the following skeleton modification, 10 g of 10 × 10 mm fragments of the alkali-treated skeleton of the sea grey urchin *Strongylocentrotus intermedius* were taken and boiled with a solution of PCaES (1 g) in toluene; then, toluene was stripped on a rotary evaporator. The product was left to dry in a vacuum oven at 60 °C until constant mass. Then, the composite was calcined at 250 °C and treated with 5% hydrochloric acid solution. After drying the acid-insoluble residue, composite 1 was obtained.

#### 2.2.6. Synthesis of Composite 2

Composite 2 was prepared similarly to composite 1 but with PCaPhS used to modify the urchin skeleton. 

### 2.3. Material Characterization Methods

The shells were washed in running water, dried, mounted on a metal sample table, and sprayed with carbon or platinum for scanning electron microscopy (SEM). The surface of the samples was imaged using SEM on a CrossBeam 1540 XV Carl Zeiss (Jena, Germany) microscope with an EDX attachment from Bruker (“Carl Zeiss”, Oberkochen, Germany). Infrared (IR) absorption spectra were recorded on a Fourier transform spectrophotometer “Spectrum: 1000” (“PerkinElmer”, Buenos Aires, Argentina). Phase identification was conducted by means of XRD on a diffractometer D8 Advance “Bruker AXS” (Bruker, Bremen, Germany), using *CuK_α_*-source, Ni-filter, angle range 10–80, scanning step 0.02°, and scanning rate 5°/min. Elemental analysis was carried out using an “EDX-7000” energy dispersive X-ray fluorescence spectrometer (“Shimadzu”, Kyoto, Japan). The thermogravimetric analysis (TGA) was carried out on a differential thermogravimetric analyser “DTG-60H” (“Shimadzu”, Kyoto, Japan); heating from 25 °C to 1000 °C with heating rate 20°/min; sample thermal analysis medium: air; a platinum crucible was used. Gel permeation chromatography (GPS) was carried out on a column with a diameter of 12 mm and a length of 100 cm. The medium was polystyrene gel with 4% divinylbenzene; particle diameter 0.1 mm; the fractions were collected in 3 mL; the content was measured using the weight method and calibrated to octaphenylsiloxane, trimethyl-, triphenylcitrosiloxane, acetylacetonate chromium, zirconium.

## 3. Results and Discussion

**Figure 1:**
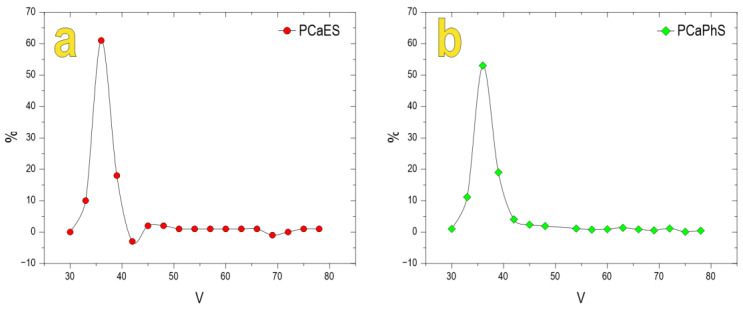
Molecular weight distribution of (**a**) PCaES and (**b**) PCaPhS.

Polycalciumorganyl sesquioxanes were obtained by reacting polyorganyl sesquioxanes [*R*SiO_1.5_]_n_ (where *R* is ethyl or phenyl radicals) with sea urchin skeleton under mechanochemical activation. The molecular weight distribution of the polymers obtained was studied by means of GPS (Figure 1). According to the GPS data, the molecular weight distribution of the polymer obtained was more than 5000 g/mol.

**Figure 2:**
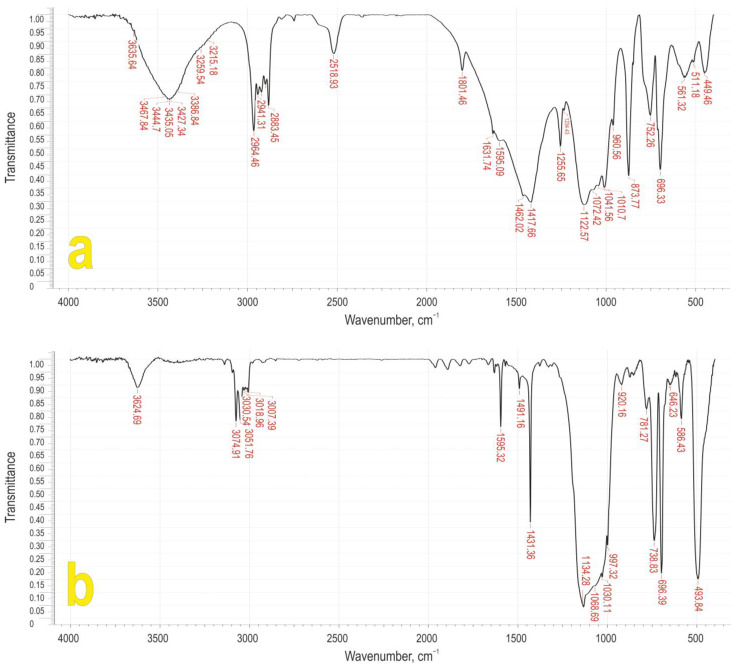
FTIR spectra of (**a**) PCaES and (**b**) PCaPhS.

The IR spectra of PCaES and PCaPhS are shown in Figure 2. The IR spectroscopy data show the presence of an intense band at 2518 cm^−1^ in PCaES and, at the same time, a band at 1010 cm^−1^ is observed, both corresponding to vibrations of the Si-C_2_H_5_ bond. In the IR spectrum of the PCaPhS, there are absorption bands at 1431, 738, and 696 cm^−1^, that correspond to vibrations of the Si-C_6_H_5_ bond. In both IR spectra, there are absorption bands of medium intensity at 3435 and 3624 cm^−1^, which correspond to the vibrations of the O-H bond in the coordinated water molecules. Several bands at 3074 and 3051 cm^−1^ correspond to the valence vibrations of the C-H bonds in aromatic radicals. Absorption bands at 1595 and 1491 cm^−1^ correspond to the valence vibrations of the C-C bond in the aromatic radical. Also observed are intense bands at 1010 and 1030 cm^−1^, corresponding to the valence vibrations of the ≡Si-O-Si≡ bond, and absorption bands at 449 and 493 cm^−1^, corresponding to the stretching vibrations of the Si-O and Ca-O bonds, respectively. Such a set of absorption bands corresponds to the vibrations of the bonding groups that are characteristic of typical polymetalorganylsiloxanes [51].

From the IR spectroscopy data, it can be concluded that the presence of absorption bands of the –COOH bond determines the cleavage reaction of the Si–O bond according to the scheme shown below:

–Ca–O–COOH + ≡Si–O–Si≡ → –Ca–O–C(O)–O–Si≡ + HO–Si≡(1)

The resulting siloxane carbonate is then hydrolysed (Equation (2)) and condensed (Equation (3)):

(≡Si–O–Ca–O)_2_C=O + 2H_2_O → 2≡Si–OH + 2HO–Ca–O–COOH, (2)

≡Si–O–Ca–OH + HO–Si≡ → ≡Si–O–Ca–O–Si≡ + H_2_O. (3)

**Figure 3:**
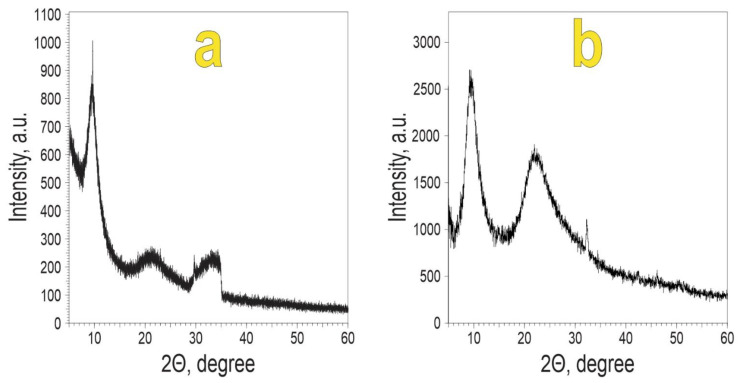
XRD data of (**a**) PCaES and (**b**) PCaPhS.

Figure 3 shows the diffractograms of polycalcium organylsesquioxanes.

It is known [52,53] that in the supramolecular structure of polymer molecules there are ordered regions where the macromolecules are stacked in a ribbon-like pattern. These regions are called coherent scattering regions (CSR). By measuring these CSR volumes, it is possible to assess the degree of order in the supramolecular structure of polymers [52]. According to [53], for mesomorphic polymers, the first reflex corresponds to the interchain distance between neighbouring atoms and the second to the intrachain distance. The volumes of the CSR and the cross-sectional areas were calculated using the Selyakov–Sherrer formula and the calculation methods proposed in [54]:

(4) $$L=\frac{0.89\cdot \lambda }{cos(\Theta )\cdot FWHM}$$

where *L*—is the size of the CSR; *λ*—is the X-ray wavelength; Ө—is the scattering angle; and *FWHM*—is the width at half-height in radians. 

The Miller–Boyer equation [53] was used to calculate the cross-sectional areas:

lg(d) = 0.61·lg(S) + 0.06,(5)

where d = d_100_; d_100_—the interplanar distance; and S—the cross-sectional area of the polymer chain.

The PCaES diffractogram corresponds to a typical mesomorphic polymetalsiloxane [41] with an interplanar distance d_100_ equal to 9.23 Å. Previously, it has been shown that d_100_ for polyvinylsiloxane is 9.0 Å, and the cross-sectional area is 78 Å^2^ [41]. The results of the calculations are given in Table 3.

The different sizes of the ethyl and phenyl radicals correspond to the difference in the d_100_ values for PCaES and PCaPhS. The cross-sectional area of the polymer chain (S) for PCaES decreases compared to the cross-sectional area of the polymer chain for PES (0.78 and 0.70 nm^2^, respectively). In contrast, the introduction of calcium atoms leads to an increase in chain spacing (1.18 and 1.08 nm^2^, respectively) for PCaPhS and PPhS. This is due to the higher stiffness and polarity of the Si-O-Si bond characteristic of PPhS as compared to the siloxane bond in PES.

The precursors for the production of PCaES and PCaPhS are more amorphous because their CSR volume is 2–3 times lower. There is a higher spatial order when calcium is introduced. In the transition from ethyl to phenyl, this is confirmed by an increase in the CSR volume. The introduction of the phenyl radical, which is more electronegative, has the effect of increasing the rigidity of the siloxane chains in comparison with the ethyl radical, which is a donor. This leads to an increase in the polarity of the bonds. The degree of crystallinity increases and the amorphousness decreases.

**Figure 4:**
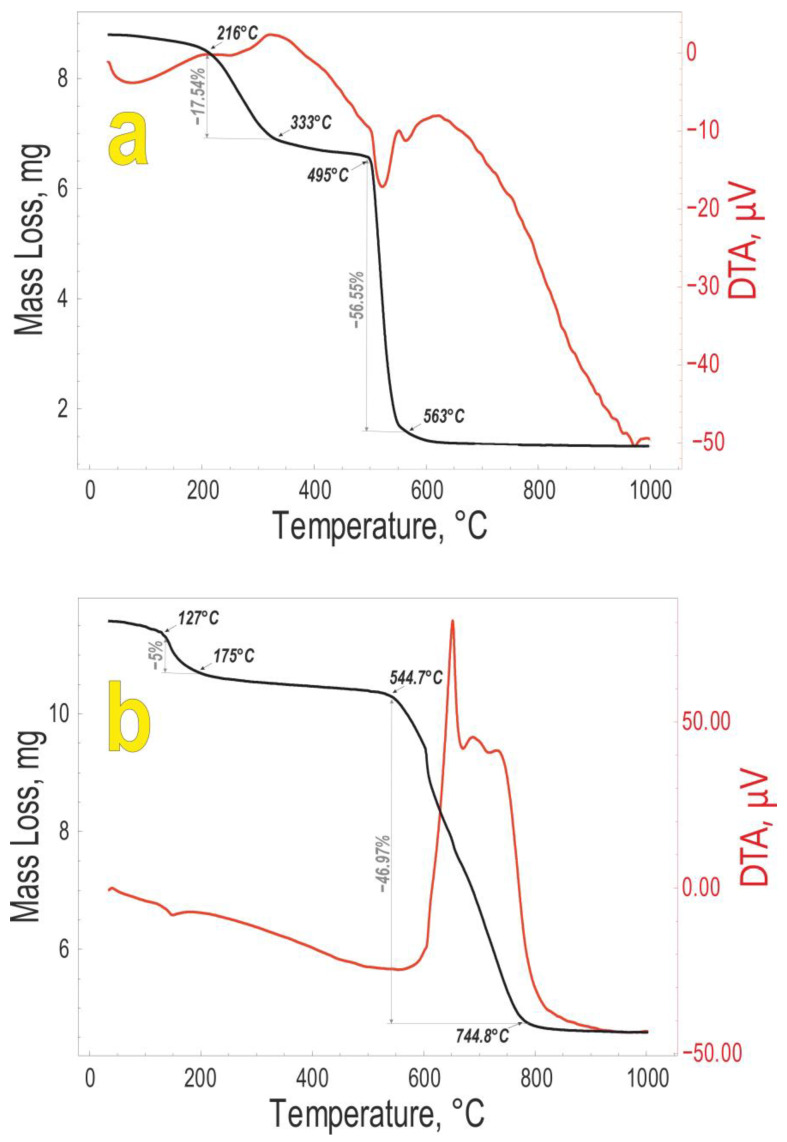
Thermogravimetric analysis of (**a**) PCaES and (**b**) PCaPhS response when heated to 1000 °C.

Figure 4 shows the TGA of the polycalcium organylsecquioxanes obtained.

The endo-effects obtained indicate the formation of cycles that are sublimated. The endo-effect is accompanied by a subsequent exo-effect. The first exo-effect observed for PCaES at 260 °C corresponds to the condensation of the hydroxyl groups and the second to the oxidation of the hydrocarbon radical. In the same way for PCaPhS, the loss of mass at 600 °C corresponds to the carbon content of the polymer.

**Figure 5:**
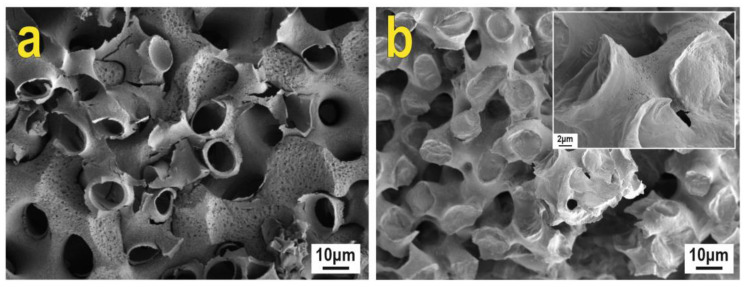
SEM images of composite surfaces: (**a**)—composite 1; (**b**)—composite 2.

Composites 1 and 2 were obtained by treating the sea urchin skeleton with polycalcium organylsesquioxanes and subsequent dissolution of the skeleton in hydrochloric acid. The microstructure of the obtained composites is a fine reticulated branched stereoma as shown by the SEM images (Figure 5). Large pores with a size of 10 μm, as well as smaller pores with a diameter in the sub-micron range, are open after processing.

The EDX data of the surface for the polycalcium organylsecquioxanes and the composites obtained on their basis are given in Table 4. The increase in the Si/Ca ratio for composite 2 compared to composite 1 is a consequence of the higher polarity of the silicon–oxygen bond. This is due to the influence of the higher electronegativity of the phenyl radical. Compared to the original PCFS, there is a destruction of the silicon–oxygen bond and an increase in the Si/Ca ratio.

The sea urchin shell is a potential source of calcium for the synthesis of calcium-containing biomaterials since the shell is mainly composed of calcium carbonate. The development of a new synthesis method using marine invertebrate skeletons and siloxanes is a very important task. The thermal stability of the introduced silicates, i.e., the ability to maintain a porous structure at high temperatures, is the key to producing an ordered porous material. The sea urchin skeleton can serve as a raw material component to source calcium ions for initiating the reaction of calcium silicate formation and play the role of a matrix for the formation of a porous inorganic composite framework; this can be a promising carrier for the targeted delivery of chemotherapeutic drugs [35].

## 4. Conclusions

The paper presents the results of polycalcium organyl sesquioxanes preparation by the reaction of polyorganyl sesquioxanes [*R*SiO_1.5_]_n_ (where *R* is an ethyl and phenyl radical) with the sea urchin skeleton under mechanochemical activation. IR spectroscopy, XRD, GPC, and TGA confirmed the composition and structure. A reaction scheme was proposed for the interaction of calcium carbonate with the siloxane bond. The reaction proceeds by cleavage of the Si-O bond, subsequent hydrolysis of the resulting siloxane carbonate, and further condensation. The introduction of calcium atoms into PPhS was found to increase the distance between the chains according to the XRD data. The transition from ethyl to phenyl radical increases the rigidity of the siloxane chains as the phenyl radical is more electronegative than the ethyl radical. The increase in the polarity of the bond and the increase in the degree of crystallinity also indicate an increase in the volume of the coherent scattering regions and an increase in the oxidation temperature of the organic radical for PCaPhS as compared to PCaES. Modification of the sea urchin skeleton by polycalcium organyl sesquioxanes in a toluene solution and treatment with hydrochloric acid to remove the sea urchin skeleton results in the destruction of the silicon–oxygen bond and an increase in the Si/Ca ratio when compared to the initial PCaPhS.

## Figures and Tables

**Table 1:** Elemental composition of polyorganylsesquioxanes.

Polymer	Found, %		Calculated, %		Mass Yield, %	Gross Formula
	C	Si	C	Si		
PES	30.9	32.2	30.6	33.6	95.4	[(C_2_H_5_SiO_1.5_)_0.97_∙0.03C_4_H_9_OH]_n_
PPhS	54.1	21.5	54.5	21.2	93.7	[C_6_H_5_SiO_1.5_∙0.17H_2_O]_n_

**Table 2:** Elemental composition of polycalcium organyl silsesquioxanes.

Polymer	Found, %			Calculated, %			Mass Yield, %	Gross Formula
	C	Si	Ca	C	Si	Ca		
PCaES	19.9	17.6	17.3	19.8	18.4	17.5	93.2	[(C_2_H_5_SiO_1.5_)_1.45_(CaO)_0.45_ (CaHCO_3_)_0.05_]∙3H_2_O
PCaPhS	37.5	21.8	16.2	34.2	21.0	15.8	65.4	[(PhSiO_1.5_)_1.2_(SiO_2_)_0.7_CaO]

**Table 3:** Results of calculating polymer chain cross-sectional areas and coherent scattering region volumes of obtained polymers from the XRD data.

Polymer	d_100_, nm	L, Å	S, nm^2^	V, Å^3^
PES	0.98	13.70	0.78	1061.75
PCaES	0.92	35.03	0.70	2447.35
PPhS	1.20	12.50	1.09	1350.00
PCaPhS	1.27	30.26	1.18	3562.71

**Table 4:** EDX data of the surface for the polycalcium organylsecquioxanes and the composites obtained on their basis.

Ratio	PCaES	PCaPhS	Composite 1	Composite 2
Si/Ca	1.5	1.9	1.33	2.4
